# Diabetes-induced hypomagnesemia is not modulated by metformin treatment in mice

**DOI:** 10.1038/s41598-018-38351-3

**Published:** 2019-02-11

**Authors:** Steef Kurstjens, Hacene Bouras, Caro Overmars-Bos, Mohamed Kebieche, René J. M. Bindels, Joost G. J. Hoenderop, Jeroen H. F. de Baaij

**Affiliations:** 1 Department of Physiology, Radboud Institute for Molecular Life Sciences, Radboud university medical center, Nijmegen, The Netherlands; 2 Faculty of Nature and Life Sciences, University of Mohammed Seddik Ben Yahia, Jijel, Algeria; 3 Faculty of Nature and Life Sciences, University of Batna 2, Batna, Algeria

## Abstract

Approximately 30% of patients with type 2 diabetes mellitus (T2D) have hypomagnesemia (blood magnesium (Mg^2+^) concentration <0.7 mmol/L). In T2D patients, treatment with metformin is associated with reduced blood Mg^2+^ levels. To investigate how T2D and metformin affect Mg^2+^ homeostasis db/m and db/db mice were treated with metformin or placebo. Mice were housed in metabolic cages to measure food and water intake, and to collect urine and feces. Serum and urinary Mg^2+^ concentrations were determined and mRNA expression of magnesiotropic genes was determined in kidney and distal colon using RT-qPCR. Db/db mice had significantly lower serum Mg^2+^ levels than db/m mice. Mild hypermagnesuria was observed in the db/db mice at two weeks, but not at four weeks. Metformin-treatment had no effect on the serum Mg^2+^ concentration and on the urinary Mg^2+^ excretion. Both in kidney and distal colon of db/db mice, there was a compensatory upregulation in the mRNA expression of magnesiotropic genes, such as transient receptor potential melastatin 6 (*Trpm6*), whereas metformin treatment did not affect gene expression levels. In conclusion, we show that T2D causes hypomagnesemia and that metformin treatment has no effect on Mg^2+^ homeostasis in mice.

## Introduction

Approximately 30% of patients with type 2 diabetes mellitus (T2D) have hypomagnesemia (blood magnesium (Mg^2+^) <0.7 mmol/L)^1,2^. Hypomagnesemia has serious clinical consequences as it increases the risk of complications such as retinopathy, nephropathy, micro and macrovascular disease and foot ulceration^3,4^. Moreover, Mg^2+^ deficiency is correlated with insulin resistance, abrogated glucose metabolism and an increased risk of developing T2D^5–7^. However, the etiology and underlying mechanisms of hypomagnesemia in T2D patients remains largely unknown^8^.

As Mg^2+^ is necessary for the activity of over 600 enzymes, it plays numerous vital physiological functions including macromolecule synthesis, energy balance and DNA transcription^9^. Moreover, Mg^2+^ stabilizes ATP and is required for its phosphor transfer reactions^10^.

The intestine and kidney collaboratively regulate Mg^2+^ balance and maintain its blood concentrations within a narrow range^11,12^. In the gut, the bulk of Mg^2+^ absorption occurs in the small intestine *via* paracellular (passive) transport^11^. In the colon, the final absorption of Mg^2+^ takes place by an active transcellular mechanism through transient receptor potential melastatin type 6/7 (TRPM6/TRPM7) cation channels^9^. In the kidney, 95–99% of filtered Mg^2+^ is reabsorbed under physiological circumstances^9^. Approximately 85% of the filtered Mg^2+^ is reabsorbed paracellularly by the proximal tubule and the thick ascending loop of Henle (TAL), where transport relies on tight junction permeability^13,14^. Active transport in the distal convoluted tubule (DCT) determines the final urinary Mg^2+^ concentration, as this is the final segment where Mg^2+^ is reabsorbed^15^. In physiological conditions, the DCT reclaims 5–10% of filtered Mg^2+^ transcellularly via TRPM6/7 channels^14,16^. The expression and/or the activity of TRPM6 is affected by SNPs, dietary Mg^2+^ intake, drugs and hormones, such as insulin and epidermal growth factor (EGF)^14,17–20^. SNPs in TRPM6 that impair its response to insulin have been associated with an increased risk of developing T2D and gestational diabetes^7,19^.

Metformin, the first-line pharmacotherapy in T2D^21^, suppresses hepatic gluconeogenesis and improves insulin sensitivity^22^. Therefore, its major clinical benefit is reducing blood glucose levels with only a minimal risk of hypoglycemia^23,24^. The most common side effects of metformin treatment are lactic acidosis, nausea and diarrhea^25^. Recent cohort studies showed that metformin use in T2D patients is associated with reduced blood Mg^2+^ levels^1,26^. However, the mechanism that underlies this correlation has not yet been elucidated. To investigate how T2D and metformin affect Mg^2+^ homeostasis, control (db/m) and diabetic (db/db) mice were treated with placebo or metformin for four weeks. Serum and urinary electrolytes were measured and mRNA expression of magnesiotropic genes was evaluated in kidney and distal colon.

## Methods

### Animal study

The animal study was approved by the animal ethics board of the Radboud University Nijmegen (RU DEC 2015-0073) and by the Dutch Central Commission for Animal Experiments (CCD, AVD103002015239). Experimental procedures were conducted in accordance with the institutional guidelines and in compliance with Dutch and European laws and policies. Twenty diabetic (db/db) and twenty control (db/m) male mice (Charles River, Germany), aged 8–10 weeks, were acclimatized for two weeks in a temperature- and light-controlled room two mice per cage (Eurostandard Type IIL), with *ad libitum* access to tap water and standard pellet chow. At day 0, diets were changed to a diet containing 0.05% (w/w) MgO (#S9074-E1107, Ssniff Spezialdiäten, GmbH, Germany) and drinking water to demineralized water. At days-2, 12 and 26 mice were housed individually in metabolic cages for 48 hours (24 hours adaptation, 24 hours collection) to measure food and water intake and to collect urine and feces. Mice were weighed twice weekly and blood was collected *via* the submandibular vein at days -2 and 15. Mice were randomly divided into four experimental groups of ten mice per group, of which half received metformin hydrochloride (0.5 mg/ml, Sigma Aldrich, MI, USA), dispersed in the drinking water. Researchers and animal caretakers were blinded for the metformin treatment. After 28 days of treatment, mice were anaesthetized by 4% (v/v) isoflurane and exsanguinated by orbital sinus bleeding, and death was confirmed by cervical dislocation. Colon and kidney tissues were cleaned with ice-cold PBS and snap-frozen in liquid nitrogen.

### RT-qPCR

TRIzol reagent (Invitrogen, Bleiswijk, the Netherlands) was used to extract total RNA from kidney and distal colon according to the manufacturer’s protocol. RNA was subjected to DNase (Promega, the Netherlands) treatment at 37 °C for 30 min and then to DNase stop buffer at 65 °C for 10 min. The RNA concentration was measured using the Nanodrop 2000c (Thermoscientific, Wilmington, DE). To synthetize cDNA, 1.5 µg of total RNA was reverse transcribed for 1 hour at 37 °C using Moloney-Murine Leukemia Virus (M-MLV) reverse transcriptase (Invitrogen, Bleiswijk, the Netherlands). SYBR Green Supermix (BioRad, Veenendaal, the Netherlands) was used to analyze the gene expression levels on a BioRad (Hercules, CA, USA) analyzer. After normalizing to housekeeping gene expression (*Hprt*), the relative gene expression was calculated by the Livak method (2^−∆∆ct^). Primers sequences are provided in Table 1.

**Table 1:** Primer sequences used for RT-qPCR.

Gene	Forward primer (5′ → 3′)	Reverse primer (5′ → 3′)
*Cldn10b*	GGAGTTCCCCTCCATGCT	GCAAAAATGGAACCGAAAAA
*Cldn14*	GTCCAGCTCCTAGGCTTCCT	CATCCACAGTCCCTTCAGGT
*Cldn16*	GTTGCAGGGACCACATTAC	GAGGAGCGTTCGACGTAAAC
*Cldn19*	GGTTCCTTTCTCTGCTGCAC	CGGGCAACTTAACAACAGG
*Cnnm4*	TCTGGGCCAGTATGTCTCTG	CACAGCCATCGAAGGTAGG
*Fxyd2*	TCAGCCTTTCTTGTGACTGG	GGTCTTCCTGTGGCCTCTACT
*Hprt*	TTGCTGACCTGCTGGATTAC	AGTTGAGAGATCATCTCCAC
*Slc12a1*	CACATGGTCTTCCACTGTGGTT	GGCTCCTCCACACAGGCTC
*Slc12a3*	CTTCGGCCACTGGCATTCTG	GATGGCAAGGTAGGAGATGG
*Slc41a1*	CATCCCACACGCCTTCCTGC	CGGCTGGCCTGCACAGCCAC
*Slc41a3*	TGAAGGGAAACCTGGAAATG	GGTTGCTGCTGATGATTTTG
*Trpm6*	CTTCACAATGAAAACCTGCCC	AAAGCCATGCGAGTTATCAGC
*Trpm7*	GGTTCCTCCTGTGGTGCCTT	CCCCATGTCGTCTCTGTCGT

*Cldn10b, claudin 10b; Cldn14, claudin 14; Cldn16, claudin 16; Cldn19, Claudin 19; Cnnm4, cyclin M4; Fxyd2, FXYD-domain containing 2; Hprt, hypoxanthine-guanine phosphoribosyltransferase; Slc12a1, solute carrier family 12 member 1; Slc12a3, solute carrier family 12 member 3; Slc41a1, solute carrier family 41 member 1; Slc41a3, solute carrier family 41 member 3; Trpm6, transient receptor potential melastatin type 6; Trpm7, transient receptor potential melastatin type 7*.

### Analytical measurements

Serum and urinary Mg^2+^ concentrations were determined using a spectrophotometric assay (Roche/Hitachi, Tokyo, Japan), according to manufacturer’s protocol. Ca^2+^ concentrations were determined by the o-cresophthalein complexone method. Absorbance for the Mg^2+^ and Ca^2+^ assays was measured at 600 nm and 570 nm, respectively, on a Bio-Rad Benchmark plus microplate spectrophotometer (Bio-Rad Laboratories, CA, USA). Serum and urinary Na^+^ and K^+^ concentrations were measured at the clinical chemistry department applying standardized methods^1^. Serum and urinary glucose concentrations were determined by a spectrophotometric assay according to the manufacturer’s protocol (Instruchemie, Delfzijl, the Netherlands).

### Statistical analyses

Interaction between the two main variables (genotype and treatment) was investigated using a two-way ANOVA test. If there was a significant interaction effect, an unpaired multiple *t* test, with the Holm–Sidak method for multiple comparisons, was used. In the absence of a significant interaction effect, a two-way ANOVA approach with a Tukey’s multiple comparisons test was used. Statistical significance was assessed using Graphpad Prism v7 (La Jolla, CA, USA, RRID: SCR_002798. A *p*-value of ≤0.05 was considered statistically significant. Results are presented as mean ± SEM.

## Results

### Metformin reduces food intake of db/db mice without affecting body weight

**Figure 1:**
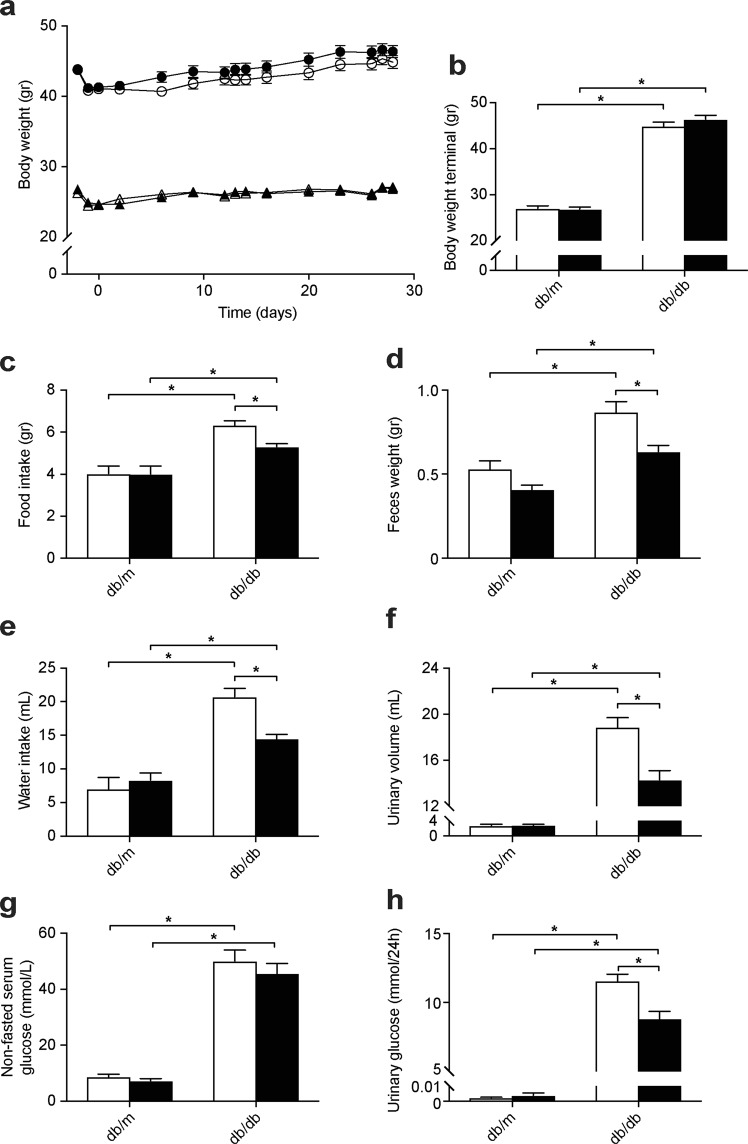
Metformin treatment does not affect body weight, but reduces food intake and urinary glucose excretion in db/db mice. Db/m and db/db mice were treated with metformin for four weeks. (**a**) Body weight of the animals, measured twice weekly and on the days of the metabolic cage experiments. Triangles, db/m mice; circles, db/db mice; open symbols, placebo-treated mice; closed symbols, metformin-treated mice. (**b**) Body weight at the end of the experiment, after four weeks of treatment. (**c**) Food intake, (**d**) total feces weight, (**e**) water intake and (**f**) urinary volume determined over a period of 24 hours, using metabolic cages, after four weeks of treatment. (**g**) Non-fasted serum glucose concentration and (**h**) 24-hour urinary glucose excretion after four weeks of treatment. Open bars, placebo-treated mice; closed bars, metformin-treated mice. Data are mean ± SEM. Depending on the absence or presence of a significant interaction effect between genotype and treatment, either a two-way ANOVA (Tukey’s multiple comparison test) or an unpaired multiple *t* test (Holm-Sidak multiple comparison test) approach, respectively, was used to determine statistical significance. *Indicates a *p* ≤ 0.05.

To investigate how T2D and its first-line treatment metformin affect Mg^2+^ homeostasis, control (db/m) and diabetic (db/db) mice were treated with metformin or placebo for four weeks. Db/db mice were significantly heavier than db/m mice (Fig. 1a,b; 27.0 ± 0.3 vs. 45.6 ± 0.6 gr. for db/m and db/db mice at four weeks, respectively, *p* ≤ 0.05). Metformin treatment had no effect on body weight in both db/m and db/db mice (Fig. 1a,b). Metformin treatment reduced the food intake only in the db/db mice (Fig. 1c). The lower food intake was accompanied by a decreased feces weight, water intake and urinary volume in the metformin-treated db/db mice (Fig. 1d–f). Metformin did not influence non-fasting serum glucose levels in both genotypes (Fig. 1g). However, the glycosuria of the db/db mice was attenuated by metformin treatment (Fig. 1h).

### Db/db mice have reduced serum Mg^2+^ concentrations

**Figure 2:**
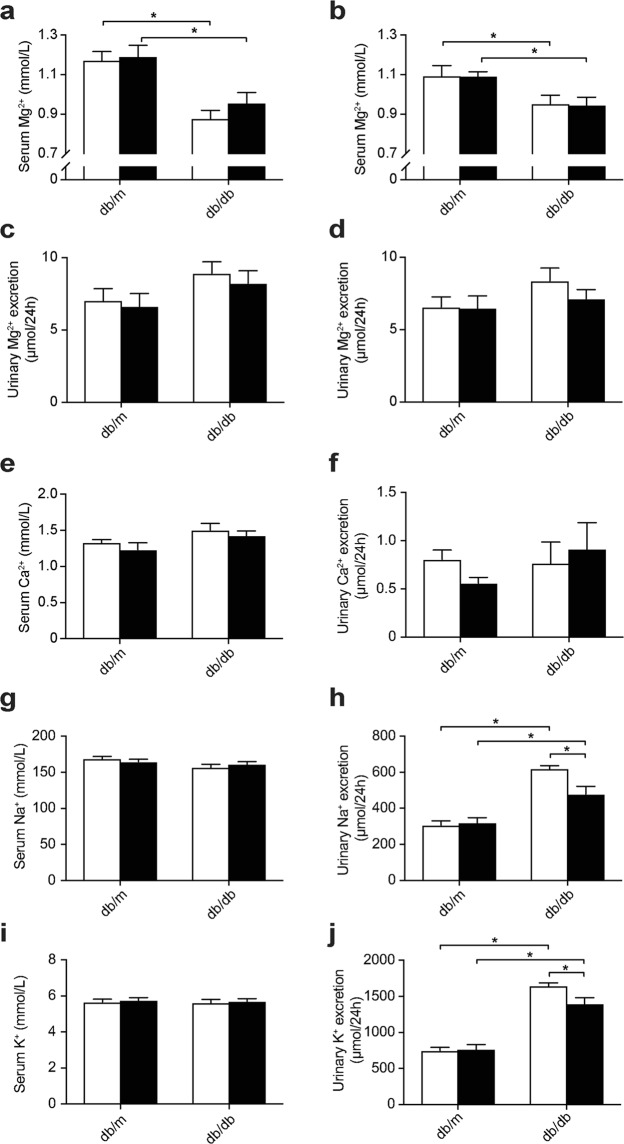
Db/db mice have a lower serum Mg^2+^ concentration which is not modulated by metformin treatment. (**a**) Serum Mg^2+^ concentration after two weeks of treatment and (**b**) after four weeks of treatment. (**c**) 24-Hour urinary Mg^2+^ excretion after two weeks of treatment (6.8 ± 0.6 vs. 8.6 ± 0.6 µmol/24 h in db/m vs. db/db mice, respectively, two-way ANOVA *p* ≤ 0.05) and (**d**) after four weeks of treatment. (**e**) Serum Ca^2+^ concentration (1.28 ± 0.05 vs. 1.46 ± 0.06 mmol/L Ca^2+^ in db/m vs. db/db mice, respectively, two-way ANOVA *p* ≤ 0.05) and (**f**) 24-hour urinary Ca^2+^ excretion, after four weeks of treatment. (**g**) Serum Na^+^ concentration (167 ± 2 vs. 159 ± 3 mmol/L Na^+^ in db/m vs. db/db mice, respectively, two-way ANOVA *p* ≤ 0.05) and (**h**) 24-hour urinary Na^+^ excretion, after four weeks of treatment. (**i**) Serum K^+^ concentration and (**j**) 24-hour urinary K^+^ excretion, after four weeks of treatment. Open bars, placebo-treated mice; closed bars, metformin-treated mice. Data are mean ± SEM. Depending on the absence or presence of a significant interaction effect between genotype and treatment, either a two-way ANOVA (Tukey’s multiple comparison test) or an unpaired multiple *t* test (Holm-Sidak multiple comparison test) approach, respectively, was used to determine statistical significance. *Indicates a *p* ≤ 0.05.

Serum Mg^2+^ concentrations were lower in db/db than db/m mice at two weeks (Fig. 2a, 1.17 ± 0.04 vs. 0.88 ± 0.04 mmol/L in db/m vs. db/db placebo-treated mice, respectively, Holm-Sidak’s multiple comparison *p* ≤ 0.05) and four weeks (Fig. 2b, 1.10 ± 0.05 vs. 0.95 ± 0.04 mmol/L in db/m vs. db/db placebo-treated mice, respectively, Holm-Sidak’s multiple comparison *p* ≤ 0.05). At two weeks, there was a significant genotype effect on urinary Mg^2+^ excretion, demonstrating an increased urinary Mg^2+^ loss in db/db mice (Fig. 2c, 6.8 ± 0.6 vs. 8.6 ± 0.6 µmol/24 h in db/m vs. db/db mice, respectively, two-way ANOVA *p* ≤ 0.05), whereas no significant difference was observed at four weeks (Fig. 2d). At four weeks, the serum Ca^2+^ concentration was higher in db/db compared to db/m mice, indicated by a significant genotype effect (Fig. 2e, 1.28 ± 0.05 vs. 1.46 ± 0.06 mmol/L Ca^2+^ in db/m vs. db/db mice, respectively, two-way ANOVA *p* ≤ 0.05). There were no significant differences on urinary Ca^2+^ excretion (Fig. 2f). Despite the higher food intake of db/db animals, a significant genotype effect demonstrated lower serum Na^+^ levels in db/db compared to db/m mice (Fig. 2g, 167 ± 2 vs. 159 ± 3 mmol/L Na^+^ in db/m vs. db/db mice, respectively, two-way ANOVA *p* ≤ 0.05). Urinary excretion of Na^+^ and K^+^ was higher in db/db than db/m mice, and metformin treatment reduced Na^+^ and K^+^ excretion only in db/db mice (Fig. 2h,j). Serum K^+^ concentrations were not different between all experimental groups (Fig. 2i).

### Db/db mice have an enhanced colonic expression of *Trpm6*

**Figure 3:**
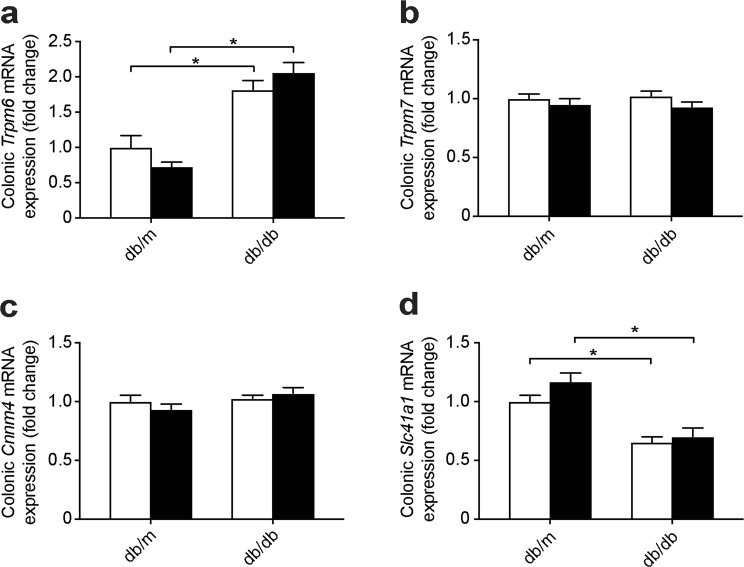
Upregulation of *Trpm6* mRNA expression in the colon of db/db mice. mRNA expression of key magnesiotropic genes in distal colon (**a**) *Trpm6*, (**b**) *Trpm7*, (**c**) *Cnnm4* and (**d**) *Slc41a1*. Open bars, placebo-treated mice; closed bars, metformin-treated mice. Data are mean ± SEM. Depending on the absence or presence of a significant interaction effect between genotype and treatment, either a two-way ANOVA (Tukey’s multiple comparison test) or an unpaired multiple *t* test (Holm-Sidak multiple comparison test) approach, respectively, was used to determine statistical significance. *Indicates a *p* ≤ 0.05.

When serum Mg^2+^ levels decrease, intestinal uptake of Mg^2+^ is enhanced^15^. Colonic mRNA expression of *Trpm6*, the major channel for regulated Mg^2+^ absorption, was elevated in db/db compared to db/m mice (Fig. 3a). There was no difference in mRNA expression of the ubiquitous Mg^2+^ channel *Trpm7* and of the Mg^2+^ transport regulator Cyclin m4 (*Cnnm4*) (Fig. 3b,c). The colonic gene expression of the basolateral Mg^2+^ transporter solute carrier family 41 (*Slc41a1*) was lower in both db/db groups (Fig. 3d).

### Db/db mice have an elevated renal expression of genes involved in Mg^2+^ handling

**Figure 4:**
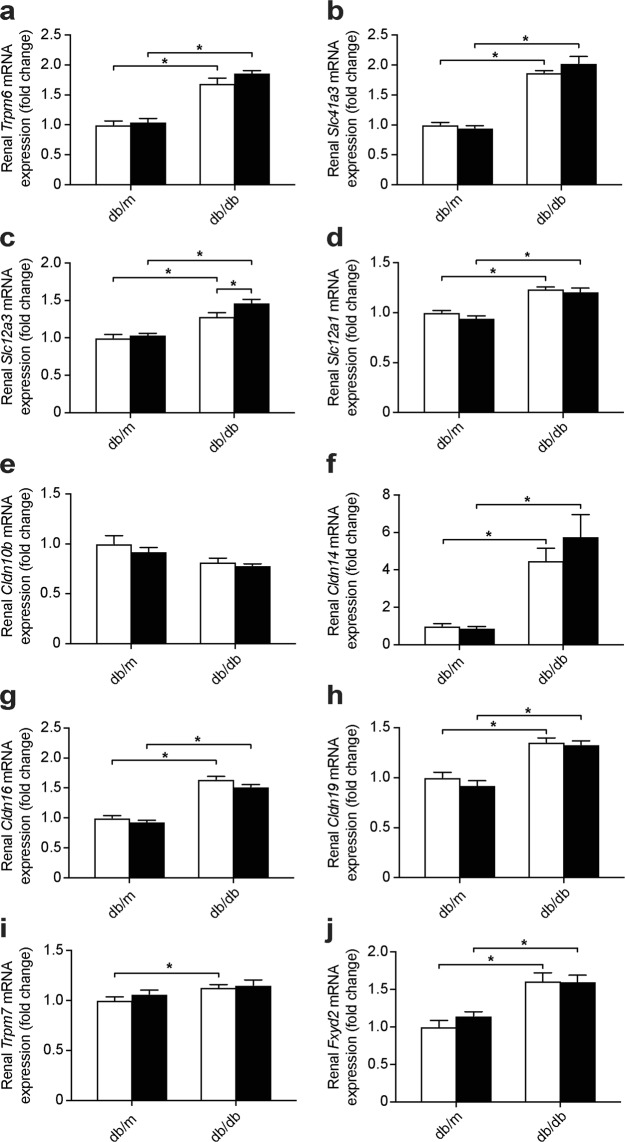
Upregulation in the expression of essential renal magnesiotropic genes in db/db mice. mRNA expression of genes involved in renal electrolyte handling (**a**) *Trpm6*, (**b**) *Slc41a3*, (**c**) *Slc12a3*, (**d**) *Slc12a1*, (**e**) *Cldn10b* (1.00 ± 0.05 vs. 0.83 ± 0.02 relative gene expression in db/m vs. db/db mice, two-way ANOVA *p* ≤ 0.05), (**f**) *Cldn14*, (**g**) *Cldn16*, (**h**) *Cldn19*, (**i**) *Trpm7* and (**j**) *Fxyd2*. Open bars, placebo-treated mice; closed bars, metformin-treated mice. Data are mean ± SEM. Depending on the absence or presence of a significant interaction effect between genotype and treatment, either a two-way ANOVA (Tukey’s multiple comparison test) or an unpaired multiple *t* test (Holm-Sidak multiple comparison test) approach, respectively, was used to determine statistical significance. *Indicates a *p* ≤ 0.05.

Db/db mice had an enhanced gene expression of the DCT-specific apical Mg^2+^ channel *Trpm6*, and the basolateral Mg^2+^ extruder *Slc41a3* (Fig. 4a,b). While both db/db groups showed a higher expression of *Slc12a3*, encoding for NCC, metformin further enhanced the expression of this gene in db/db mice (Fig. 4c). The driving force for paracellular Mg^2+^ uptake in the TAL is generated by NKCC2, encoded by *Slc12a1*, which is expressed higher in db/db mice (Fig. 4d). A significant genotype effect indicated a decreased expression of *Claudin 10b* (*Cldn10b*) in db/db mice (Fig. 4e, 1.00 ± 0.05 vs. 0.83 ± 0.02 relative gene expression in db/m vs. db/db mice, two-way ANOVA *p* ≤ 0.05). In contrast, the mRNA expression of *Cldn14*, *Cldn16* and *Cldn19* was enhanced in db/db mice (Fig. 4f–h). The gene expression of the ubiquitous Mg^2+^ channel *Trpm7* was elevated in the placebo-treated db/db mice and the expression of *Fxyd2*, encoding for the gamma subunit of the Na^+^-K^+^-ATPase, was enhanced in both db/db groups (Fig. 4i,j).

## Discussion

Hypomagnesemia is a common clinical feature in T2D patients^1,3^. Metformin use is associated with a lower blood Mg^2+^ concentration in these patients^1,26^. In this study, db/db mice developed hypomagnesemia with compensatory upregulation of key renal and colonic magnesiotropic genes. Metformin treatment had no effect on Mg^2+^ homeostasis in either control or diabetic mice. Our data demonstrate that hypomagnesemia is a consequence of T2D and is not modulated by metformin treatment in mice.  INSERT ENTER Metformin is the first-line therapy for T2D^27^. In large-scale observational cohort studies metformin-use is associated with lower serum Mg^2+^ levels and reduced renal Mg^2+^ wasting in T2D patients^1,26,28–30^. In a small intervention study in T2D patients, metformin treatment resulted in a minor reduction in the serum Mg^2+^ concentration (from 0.72 to 0.70 mmol/L), despite major improvements in the blood glucose concentration^29^. In our study, metformin treatment did not affect the serum Mg^2+^ concentration and urinary Mg^2+^ excretion in db/db and db/m mice. In addition, metformin did not alter gene expression of colonic and renal Mg^2+^ transporters. This is in line with a study that observed no effect of a two-week metformin treatment on serum Mg^2+^ levels in streptozotocin-induced diabetic rats^31^. Possibly, a two- to four-week treatment duration is too short to detect effects on Mg^2+^ homeostasis. The association between metformin and lower serum Mg^2+^ levels in T2D patients could also be caused by other factors that were not included in the analyses. For instance, a well-known side effect of metformin-treatment is chronic diarrhea, leading to intestinal malabsorption and hypomagnesemia^28^.

Hypomagnesemia is prevalent in over 30% of T2D patients^32–35^. A remaining question is whether hypomagnesemia is the cause or the consequence of T2D^8^. In the present study, db/db mice developed hypomagnesemia, indicating that hypomagnesemia is a consequence of T2D. At the fourth week of the experiment, db/db mice developed massive glycosuria but no renal Mg^2+^ wasting. This finding is against the leading hypothesis that renal Mg^2+^ wasting in T2D patients is a result of glycosuria^2,3,36^. Indeed, metformin treatment noticeably decreased glycosuria in db/db mice but did not modify the urinary Mg^2+^ excretion. This is in line with recent observations that glycosuria-causing SGLT2 inhibitors, lead to a mild increase in serum Mg^2+^ levels^37,38^. Therefore, it is unlikely that glycosuria underlies hypermagnesuria-induced hypomagnesemia in T2D. As db/db mice develop severe hyperinsulinemia, the observed hypomagnesemia could be a consequence of a Mg^2+^-shift towards the intracellular compartment, induced by insulin^39–41^. Future studies should focus on measuring intracellular Mg^2+^ concentrations in diabetic mice.

The kidneys are essential in maintaining the serum Mg^2+^ concentration within the physiological range^15^. The DCT is the final segment where Mg^2+^ can be reabsorbed^9^. In the DCT, regulated Mg^2+^ reabsorption takes place transcellularly *via* TRPM6^18^. Mg^2+^ uptake by TRPM6 is dependent on NCC, although the underlying mechanism remains largely unknown^42,43^. Gene expression levels of *Trpm6* and *Slc12a3*, encoding for NCC, were enhanced in db/db mice, indicative of compensation in the DCT. As only a minor hypermagnesuria is observed at two-weeks, and no hypermagnesuria at four-weeks, there appears to be proper renal compensation in the db/db mice. The TAL is responsible for the bulk of renal Mg^2+^ reabsorption^9^. In the TAL, paracellular Mg^2+^ and Ca^2+^ reabsorption is regulated by the Cldn14/16/19 complex^44,45^. *Cldn14* mRNA expression is strongly regulated by dietary Ca^2+^ intake^46,47^. The high food intake, and therefore high Ca^2+^ intake, of db/db mice is likely the underlying cause of the extensive upregulation of *Cldn14* expression. The high expression of *Cldn14* will have a negative effect on Mg^2+^ reabsorption in the TAL, leading to a compensatory increase in *Cldn16/19* expression^48^. In contrast, gene expression of *Cldn10b* was decreased. Cldn10b enhances the Na^+^-permeability of the TAL, and thereby indirectly increases uptake of Mg^2+^ and Ca^2+^ in the TAL. Therefore, *Cldn10b*-deficient mice develop hypermagnesemia and hypomagnesuria. Likely, the observed reduction in *Cldn10b* expression in the db/db mice is a response to the high osmolality of the pro-urine. INSERT ENTER The strength of this study is that using oral metformin treatment in diabetic mice closely resembles the human situation. Db/db mice developed hypomagnesemia making them an excellent model to study the mechanisms of hypomagnesemia in T2D. Moreover, this study extensively investigated differences in expression of all known genes involved in Mg^2+^ transport, in both kidney and colon. Some limitations have to be considered. The fact that metformin treatment did not affect Mg^2+^ homeostasis raises the question whether the dose and duration of metformin treatment were sufficient. However, the metformin treatment reduced the food intake of db/db mice, a known positive effect of metformin. Moreover, the dosage of metformin that the db/db received (0.5 mg/ml, equivalent to a daily intake of approximately 165 mg/kg bodyweight) is similar to previous studies investigating the metabolic effects of metformin in mice^49–52^. A second limitation is that the expression of genes such as *Cldn10b/14*, *Slc12a1* and *Slc12a3* is regulated by both dietary intake and serum levels of K^+^, Na^+^ and Ca^2+^ ^43,44,53^. As db/db mice have hyperphagia, their dietary intake of ions is also increased. Despite the higher food intake, db/db mice still develop hypomagnesemia. However, for other differences between db/m and db/db mice it is difficult to differentiate whether they are caused by T2D-related factors or by a higher food intake.

In conclusion, hypomagnesemia is a consequence of T2D, which is not affected by metformin treatment. The reason that metformin-users have lower serum Mg^2+^ concentrations is likely mediated by other factors, and not by a direct effect of metformin on Mg^2+^ (re)absorption.

## Data Availability

The datasets generated during and/or analysed during the current study are available from the corresponding author on reasonable request.
